# Implementation research: towards universal health coverage with more doctors in Brazil

**DOI:** 10.2471/BLT.16.178236

**Published:** 2017-02-01

**Authors:** Leonor Maria Pacheco Santos, Aimê Oliveira, Josélia Souza Trindade, Ivana CHC Barreto, Poliana Araújo Palmeira, Yamila Comes, Felipe OS Santos, Wallace Santos, João Paulo Alves Oliveira, Vanira Matos Pessoa, Helena Eri Shimizu

**Affiliations:** a Universidade de Brasília, Faculdade de Ciências da Saúde, Departamento de Saúde Coletiva, Campus Universitário Darcy Ribeiro, Brasília, DF, 70910-900 Brasília, Brazil.; b Fundação Osvaldo Cruz – Ceará, Torre Saúde, Fortaleza, Brazil.; c Universidade Federal de Campina Grande, Centro de Educação e Saúde, Cidade Universitária, Cuité, Brazil.; d Fundação Osvaldo Cruz – Brasília, Campus Universitário Darcy Ribeiro, Brasília, Brazil.; e Secretaria de Estado de Saúde do Distrito Federal, Diretoria Regional de Atenção Primária – DIRAPS, Brasília, Brazil.

## Abstract

**Objective:**

To evaluate the implementation of a programme to provide primary care physicians for remote and deprived populations in Brazil.

**Methods:**

The *Mais Médicos* (More Doctors) programme was launched in July 2013 with public calls to recruit physicians for priority areas. Other strategies were to increase primary care infrastructure investments and to provide more places at medical schools. We conducted a quasi-experimental, before-and-after evaluation of the implementation of the programme in 1708 municipalities with populations living in extreme poverty and in remote border areas. We compared physician density, primary care coverage and avoidable hospitalizations in municipalities enrolled (*n* = 1450) and not enrolled (*n* = 258) in the programme. Data extracted from health information systems and Ministry of Health publications were analysed.

**Findings:**

By September 2015, 4917 physicians had been added to the 16 524 physicians already in place in municipalities with remote and deprived populations. The number of municipalities with ≥ 1.0 physician per 1000 inhabitants doubled from 163 in 2013 to 348 in 2015. Primary care coverage in enrolled municipalities (based on 3000 inhabitants per primary care team) increased from 77.9% in 2012 to 86.3% in 2015. Avoidable hospitalizations in enrolled municipalities decreased from 44.9% in 2012 to 41.2% in 2015, but remained unchanged in control municipalities. We also documented higher infrastructure investments in enrolled municipalities and an increase in the number of medical school places over the study period.

**Conclusion:**

Other countries having shortages of physicians could benefit from the lessons of Brazil’s programme towards achieving universal right to health.

## Résumé

**Objectif:**

Évaluer la mise en œuvre d'un programme destiné à renforcer la présence de médecins généralistes auprès des populations isolées et défavorisées du Brésil.

**Méthodes:**

Dans le cadre du programme *Mais Médicos* (plus de médecins), qui a débuté en juillet 2013, des appels publics ont été lancés afin de recruter des médecins dans les zones prioritaires. D'autres stratégies ont consisté à augmenter les investissements dans les infrastructures de soins primaires et à ouvrir plus de places dans les écoles de médecine. Nous avons réalisé une évaluation quasi-expérimentale, avant-après, de la mise en œuvre du programme dans 1708 municipalités dont certaines populations vivaient en situation d'extrême pauvreté ainsi que dans des zones frontalières reculées. Nous avons comparé la densité de praticiens, la couverture de soins primaires et le nombre d'hospitalisations évitables dans les municipalités qui participaient au programme (*n* = 1450) et celles qui n'y participaient pas (*n* = 258). Des données extraites de systèmes d'information sanitaire et de publications du ministère de la Santé ont été analysées.

**Résultats:**

Au mois de septembre 2015, 4917 médecins étaient venus s'ajouter aux 16 524 praticiens déjà en place dans des municipalités où vivaient des populations isolées ou défavorisées. Le nombre de municipalités comptant ≥ 1,0 médecin pour 1000 habitants avait doublé, passant de 163 en 2013 à 348 en 2015. La couverture de soins primaires dans les municipalités participantes (sur la base de 3000 habitants par équipe de soins primaires) était passée de 77,9% en 2012 à 86,3% en 2015. Le nombre d'hospitalisations évitables dans les municipalités participantes était passé de 44,9% en 2012 à 41,2% en 2015, mais était resté identique dans les municipalités témoins. Sur la période étudiée, nous avons également observé une hausse des investissements dans les infrastructures dans les municipalités participantes ainsi qu'une augmentation du nombre de places dans les écoles de médecine.

**Conclusion:**

D'autres pays qui souffrent d'une pénurie de médecins pourraient s'inspirer des leçons du programme brésilien pour garantir le droit de tous à la santé.

## Resumen

**Objetivo:**

Evaluar la implementación de un programa que ofrezca médicos de atención primaria para poblaciones remotas y desfavorecidas de Brasil.

**Métodos:**

En julio de 2013 se lanzó el programa *Mais Médicos *(Más Médicos), el cual hizo un llamamiento público para contratar médicos para zonas prioritarias. Otras estrategias fueron aumentar las inversiones en infraestructuras de atención primaria y proporcionar más plazas en las escuelas de medicina. Se realizó una evaluación cuasiexperimental antes y después de la implementación del programa en 1 708 municipios con una población en extrema pobreza, así como en zonas fronterizas remotas. Se comparó la densidad de médicos, la cobertura de atención primaria y las hospitalizaciones evitables en los municipios inscritos (*n *= 1 450) y no inscritos (*n *= 258) en el programa. Se analizaron los datos recopilados de los sistemas de información sanitaria y de publicaciones del Ministerio de Sanidad.

**Resultados:**

En septiembre de 2015, se sumaron 4 917 médicos a los 16 524 que ya se encontraban en municipios con poblaciones remotas y desfavorecidas. El número de municipios con ≥ 1,0 médico por cada 1 000 habitantes se duplicó, de 163 en 2013 a 348 en 2015. La cobertura de atención primaria en municipios inscritos (basada en 3 000 habitantes por equipo de atención primaria) aumentó de un 77,9% en 2012 a un 86,3% en 2015. Las hospitalizaciones evitables en municipios inscritos se redujeron de un 44,9% en 2012 a un 41,2% en 2015, pero permanecieron sin cambios en los municipios de control. También se documentaron mayores inversiones en infraestructuras en los municipios inscritos y un aumento en el número de plazas de las escuelas de medicina durante el periodo de estudio.

**Conclusión:**

Otros países con escasez de médicos podrían beneficiarse de las lecciones del programa brasileño para conseguir un derecho universal a la sanidad.

## ملخص

الغرض تقييم تنفيذ برنامج لتوفير الأطباء المقدمين لخدمات الرعاية الصحية الأولية للقطاعات السكانية المحرومة في المناطق النائية في البرازيل.

الطريقة بدأ العمل ببرنامج *Mais Médicos *****(المزيد من الأطباء) في تموز/يوليو 2013 بدعوات شعبية لتعيين أطباء في المناطق ذات الأولوية. وتمثلت الاستراتيجيات الأخرى في زيادة الاستثمارات في البنية التحتية المرتبطة بالرعاية الصحية الأولية وإتاحة المزيد من الأماكن في كليات الطب. وأجرينا تقييمًا شبه تجريبيًا قبليًا وبعديًا يتعلق بتنفيذ البرنامج، وذلك في 1708 إقليم يضم جماعات سكانية تحيا في فقر مدقع وتقطن في مناطق حدودية نائية. وقارنا بين الأقاليم المسجلة ضمن البرنامج (العدد = 1450) والأقاليم غير المسجلة بالبرنامج (العدد = 258) من حيث كثافة عدد الأطباء ومدى تغطية الرعاية الصحية الأولية وحالات العلاج بالمستشفيات الذي كان من الممكن تجنبه. وتم تحليل البيانات المستمدة من نظم المعلومات الصحية ومطبوعات وزارة الصحة.

النتائج بحلول أيلول/سبتمبر 2015، تمت إضافة 4917 طبيب إلى الأطباء الذين بلغ عددهم 16,524 من الذين كانوا عاملين بالفعل في الأقاليم التي تضم جماعات سكانية محرومة بالمناطق النائية. وتضاعف عدد الأقاليم التي ضمت ما يزيد عن أو يساوي 1.0 طبيب لكل 1000 من السكان، حيث كان عدد الأطباء 163 طبيبًا في عام 2013 ووصل إلى 348 طبيبًا في عام 2015. وزاد مدى تغطية خدمات الرعاية الصحية الأولية في الأقاليم المسجلة بالبرنامج (القائمة على 3000 من السكان لكل فريق من فرق الرعاية الصحية الأولية) من 77.9‏% في عام 2012 ليصل إلى 86.3‏% في عام 2015. وانخفض عدد حالات العلاج بالمستشفيات الذي كان من الممكن تجنبه في الأقاليم المسجلة بالبرنامج من ‏44.9% في عام 2012 إلى ‏41.2% في عام 2015، ولكنه ظلت دون أي تغير في الأقاليم التي تمثل فئة الشاهد. كما سجلنا زيادةً في استثمارات البنية التحتية في الأقاليم المسجلة بالبرنامج وارتفاعًا في عدد الأماكن المتاحة في كليات الطب على مدى فترة الدراسة.

الاستنتاج يمكن للبلدان الأخرى التي تعاني من نقص في عدد الأطباء الانتفاع من الدروس المستفادة من البرنامج الذي تم تطبيقه في البرازيل سعيًا لتوفير الحق في التمتع بالصحة.

## 摘要

**目的:**

旨在评估一项为巴西偏远地区和贫困地区人群提供初级护理医师计划的实施情况。

**方法:**

*伴随着公众为重点地区招募医师的呼声,Mais Médicos* (扩大医生队伍)计划于 2013 年 7 月启动。 也采取了其他策略,以增加初级护理基础设施投资并扩大医学院招生计划。 我们对该计划在 1708 个地区的实施情况进行了准实验、前后对比评估,这些地区的居民生活在极端贫困的区域以及偏远边境区域。 我们对参与地区 (*n* = 1450) 和未参与地区 (*n* = 258) 的医师密度、初级护理覆盖率以及可避免住院率进行了比较。 我们对从卫生信息系统和卫生部出版物中提取的数据进行了分析。

**结果:**

截止 2015 年 9 月,在偏远和贫困地区,在原有的 16 524 名医师的基础上新增了 4917 名医师。 从 2013 年的 163 到 2015 年的 348,每 1000 名居民配有 1.0 医生的地区的数量增加了一倍。参与地区的初级护理覆盖率(基于每个初级护理团队 3000 名居民)从 2012 年的 77.9% 增至 2015 年的 86.3%。参与地区的可避免住院率从 2012 年的 44.9% 降至 2015 年的 41.2%,但对照地区的可避免住院率保持不变。 我们也证实,在调查期间,参与地区加大了基础设施投资且扩大了医学院招生计划。

**结论:**

其他存在医师短缺问题的国家可以学习巴西实现全民健康权利计划的经验并从中受益。

## Резюме

**Цель:**

Оценить реализацию программы по направлению врачей общей практики для оказания первичной медико-санитарной помощи бедным группам населения и группам, живущим в отдаленных районах Бразилии.

**Методы:**

Программа *Mais Médicos* (Больше врачей) была начата в июле 2013 года с открытого конкурса для врачей в приоритетных областях. Другие стратегии включали увеличение объема инвестиций в инфраструктуру первичной медико-санитарной помощи и обеспечение большего количества мест в медицинских учебных заведениях. Мы провели квазиэкспериментальную оценку реализации программы перед началом и после ее окончания в 1708 муниципалитетах с населением, живущим в условиях крайней нищеты и в отдаленных приграничных районах. Мы сравнили плотность врачей, охват первичной медико-санитарной помощью и количество предотвратимых случаев госпитализации в муниципальных образованиях, включенных (*n* = 1450) и не включенных (*n* = 258) в программу. Были проанализированы данные, извлеченные из информационных систем здравоохранения и публикаций Министерства здравоохранения.

**Результаты:**

По состоянию на сентябрь 2015 года к 16 524 врачам, уже работающим в муниципалитетах с бедными группами населения и группами, живущими в отдаленных районах, добавилось 4917 врачей. Число муниципалитетов с ≥1,0 врачей на 1000 жителей увеличилось в два раза: с 163 в 2013 году до 348 в 2015 году. Охват первичной медико-санитарной помощью во включенных муниципальных образованиях (3000 жителей на команду по оказанию первичной медицинской помощи) увеличился с 77,9% в 2012 году до 86,3% в 2015 году. Количество предотвратимых случаев госпитализации во включенных муниципалитетах снизилось с 44,9% в 2012 году до 41,2% в 2015 году, но осталось неизменным в муниципалитетах сравнения. Мы также зарегистрировали более высокие инвестиции в инфраструктуру во включенных муниципалитетах и увеличение мест в медицинских учебных заведениях в течение периода исследования.

**Вывод:**

Другие страны, страдающие от дефицита врачей, могут извлечь пользу из программы Бразилии на пути к обеспечению всеобщего права на охрану здоровья.

## Introduction

Brazil’s large territory, partially covered by dense forests, is a challenge for efforts to achieve universal health access and coverage for the population. After the re-establishment of democracy and the new Constitution in 1988, access to health care was established as a constitutional right and an obligation of the state. A unified national health system – the *Sistema Único de Saúde* – was founded in 1988,^1^ based on the principles of universal, integrated health care with equality of access to all. Financed with public funds, the national health system provides preventive and curative health care free of charge.

Although the health-care system has made major advances in the past 25 years, such as enhancing the coverage of antenatal care and measles vaccination,^2^ transforming the constitutional right into a reality still remains a challenge.^1^ For example, a study by the federal medical council in October 2012^3^ showed that there were 388 015 physicians for an estimated population of 191 million people.^4^ This average density of 2.00 physicians per 1000 inhabitants nationwide varied from 0.71 physicians per 1000 people in the state of Maranhão (the poorest state) to 4.09 per 1000 people in the Federal District (the wealthiest state).^3^ Distribution within the states was also unequal; some municipalities in the north and north-east semi-arid states had no physicians.^5^ The causes of these inequalities of distribution are related to problems of recruitment and retention of physicians at basic health units in the national health system.^6^^–^^8^ These include job insecurity and low career prospects in the public health system; poor working conditions and infrastructure in primary care facilities; and better job opportunities elsewhere.

Brazil needs an equitable distribution of the health workforce, particularly physicians, if it is to achieve the sustainable development goals (SDGs) on health and equality (SDGs 3 and 10).^9^^,^^10^ The tendency for physicians to concentrate in urban centres is a problem that affects many countries. Strategies for retaining health-care professionals in areas of vulnerability range from providing incentives and support, to enforcement measures such as mandatory civil service.^11^^–^^15^ To increase the number of primary health-care physicians for priority areas the Brazilian government set up the *Mais Médicos* (More Doctors) programme in July 2013. This employed strategies aligned with those proposed by the World Health Organization to improve the retention of health workers in remote and rural areas.^16^^,^^17^

The aim of the present study was to describe the implementation of the programme in remote and deprived areas in Brazil and to evaluate its contribution to progress towards achieving universal health access and coverage for these vulnerable populations.

## Methods

### Intervention

The *Mais Médicos* programme aimed to actively recruit primary care physicians and provide incentives to retain them through better working conditions and increased investment in the primary care infrastructure. The programme also addressed the shortage of physicians in the mid- and long-term through an expansion of medical training.

All 5570 municipalities of Brazil were eligible to enrol in the first strand of the programme: the emergency provision of physicians for primary health care. Priority was given to municipalities with 20% or more of the population in extreme poverty; in deprived areas of state capitals and metropolitan regions; in rural and remote regions, e.g. the north-east semi-arid region and the north (Amazon) region; and with traditional populations, such as Maroon communities. All the 34 indigenous health districts that provide primary health care to 517 383 Brazilian indigenous people in forest and rural areas (2010 Census data) were prioritized.^18^ The programme offers physicians incentives to enrol, with three-year work contracts, competitive salaries (compatible to market values), lodging expenses and airline tickets to home once a year.

The second strand of the programme was to enhance the quality of primary health care and guarantee better working conditions for health professionals by investing in the infrastructure of basic health units. Municipalities could apply to the health ministry for funds to construct or refurbish health units or to purchase basic equipment such as refrigerators, autoclaves, otoscopes and sphygmomanometers.

The third strand was to increase the overall number of physicians in Brazil by providing more undergraduate medical school places in states where there were ≤ 1.5 places per 10 000 population or ≤ 2.7 doctors per 1000 population.^19^ The federal government planned to authorize 11 447 new undergraduate medical course places by 2017. Priority was initially for towns with a population of 70 000 inhabitants or more and without a medical school in their territory;^20^ later this changed to areas 75 km away from any medical school.^21^ The programme also made changes in the national curriculum to tailor undergraduate medical training to the national unified health system.^22^

### Implementation

The programme was launched nationwide via a provisional presidential decree on 8 July 2013 and was enacted into Law No. 12.871 of 22 October 2013. Public calls for enrolment of municipalities and recruitment of physicians were opened on the health ministry website (http://www.maismedicos.gov.br). From 8 July 2013 to 2 October 2015 there were six public calls for municipalities to join and eight public calls for physicians to enrol.

At the first public call, in July 2013, 3511 of the 5570 municipalities in Brazil enrolled in the programme and requested 15 460 physicians. Although the programme prioritized the recruitment of Brazilian physicians, only 1096 nationals spontaneously registered and were hired, while another 522 positions were occupied by physicians from other countries where the ratio of physicians to inhabitants was higher than in Brazil.^23^ Both Brazilians and the individual exchange foreign professionals could indicate up to six locations of choice. With the assistance of the Pan American Health Organization, an agreement was signed with the Cuban government to supply physicians with primary health-care experience.^19^ Cuban physicians were sent to priority municipalities, usually remote places, which had not been chosen by Brazilian doctors. The health ministry licensed physicians who obtained their diplomas abroad to practise medicine for three years only in the assigned municipality.

In November 2013, according to the health minister, all the regions including rural communities and the Brazilian semi-arid region had at least one doctor.^5^ By September 2015 the programme had increased to 4004 participating municipalities and provided 17 625 physicians: 11 329 (64.3%) Cubans, 5600 (31.8%) Brazilians and 696 (3.9%) professionals from other countries.

### Study design

We conducted a quasi-experimental, before-and-after evaluation of the implementation of the programme in 1708 priority municipalities with remote and deprived populations. These were municipalities with 20% or more of the population living in extreme poverty and those located in the country border areas. The proportion of the population in extreme poverty in each municipality was obtained from the Ministry of Social Development website.^24^ The cut-off for defining social vulnerability due to extreme poverty in July 2013 was family monthly income per capita below 70 Brazilian reais (equivalent to 19.4 United States dollars, US$).

We compared the 1450 remote and deprived municipalities that enrolled in 2013 or 2104 and received physicians, with the 258 municipalities that were eligible and prioritized but did not enrol in the programme. Of the original 1450 enrolled municipalities, 39 (2.7%) remained in the programme for two years but dropped out in 2015 and so were lost to follow-up.

The study was approved by the research ethics board of the University of Brasilia (protocol no. 399.461, CAAE 21688313.9.0000.0030).

### Outcomes and data sources

We extracted data on outcomes of the programme in the years before and after implementation of the programme in the studied municipalities.

The number of physicians in each municipality in April 2013, just before the programme started, was obtained from the Brazilian National Register of Health Institutions Establishments.^25^ The number of physicians allocated by the programme in September 2015 was requested by the health ministry.^19^ For this study we included physicians from *Mais Médicos* and from PROVAB (*Programa de Valorização da Atenção Básica*), a similar programme,which attracted fewer physicians and only Brazilians. In January 2015 the health ministry merged the two programmes.^19^

We estimated the density of physicians, i.e. the number of physicians per 1000 inhabitants, using official population estimates based on Brazilian national census data.^4^ The density of physicians across Brazil was then mapped in five bands: ≥ 1.0, ≥ 0.7 to < 1.0, ≥ 0.4 to < 0.7, ≥ 0.1 to < 0.4, and < 0.1 physicians per 1000. The health ministry defined adequate density as at least 1.0 physician per 1000 inhabitants. Vector maps were created in *Quantum* geographical information system software, version 2.12.0 (Open Source Geospatial Foundation; http://www.osgeo.org/).

The health ministry stipulates that each family-health team covers 3000 people. To estimate primary health-care coverage, we obtained data on the number of primary health-care teams and the population in each studied municipality over the period 2011–2015. The number of teams was multiplied by 3000, divided by the number of inhabitants and multiplied by 100. Data were extracted from the health ministry’s DATASUS database.^26^

We estimated the rate of avoidable hospitalizations in each studied municipality over the period 2011–2015 from the number of hospitalizations due to ambulatory care-sensitive conditions as a percentage of the total number of clinical admissions. In Brazil 17 ambulatory care-sensitive conditions were defined in the Ministry of Health Directive MS/SAS No. 221 of 17 April 2008. Ambulatory care-sensitive conditions are those which are potentially preventable through early diagnosis, treatment of acute conditions or control and monitoring of chronic diseases.^27^ Data were obtained from the health ministry website.^26^

It was not possible to calculate confidence limits because we employed aggregated data.

We estimated the annual expenditure in Brazilian real on construction of new basic health units and renovation of existing units in the different regions of the country over the period 2012–2015. Data were obtained from the health ministry’s Support Unit for Strategic Management database.^28^ On 31 May 2016, the conversion rate was 1 Brazilian real to US$ 0.28.

To describe trends in medical school education we obtained data on the number of medical schools and the number of undergraduate medical places per 10 000 inhabitants in the different regions over the period 1994–2015. These data were collected from the medical schools of Brazil website^29^ and from the health ministry.^19^

## Results

### Distribution of municipalities

**Fig. 1:**
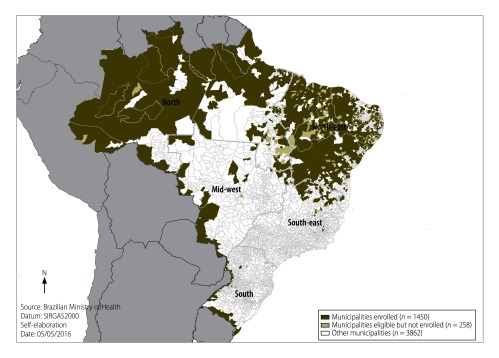
**Municipalities with remote and deprived populations according to enrolment in the *Mais Médicos* programme in Brazil, 2013–2015**

Notes: The *Mais Médicos* programme was implemented in July 2013. Municipalities with remote and deprived populations were those with 20% or more of the population living in extreme poverty (per capita family income below 70 Brazilian reais, equivalent to 19.4 United States dollars) in 2013 or those in the border areas. Enrolled municipalities were those that joined the programme and received physicians allocated by the programme. Of the 1450 municipalities enrolled in the programme in 2013–14, 39 (2.7%) had dropped out by September 2015. The 258 municipalities not enrolled were eligible for the programme but never enrolled at the time of the study. Other municipalities were not included in the study.

Sources: Secretariat of Labour Management and Health Education and the Department of Planning and Regulation of the Provision of Health Care Professionals, Brazilian Ministry of Health, on request, 2015.

Fig. 1 shows the geographical distribution of the 1708 studied municipalities with remote or deprived populations. These were mainly concentrated in the north-east and north of the country (Amazon region) or in the mid-west and south regions at the borders. Fig. 1 also maps the distribution of the 1450 (84.9%) municipalities that enrolled in 2013–14 and were allocated physicians by the programme and the 258 municipalities eligible and prioritized, but not enrolled in the programme in the period 2013–2015.

### Density of physicians

By September 2015, 4917 physicians had been allocated by the programme to the municipalities with remote and deprived populations. They accounted for 27.9% of the total of 17 625 physicians enrolled in the whole programme. Their nationalities were 3566 from Cuba (72.5%), 1270 from Brazil (25.8%) and 81 (1.6%) from other countries. Table 1 shows the numbers of municipalities with remote and deprived populations in each region of Brazil and the number and percentage of physicians allocated by the programme to these municipalities. The highest number of physicians was allocated to the north-east (3547, 72.2%), followed by the north (952, 19.4%).

**Table 1:** Enrolment of municipalities with remote and deprived populations in the *Mais Médicos* programme, and physicians allocated to these municipalities, by region, Brazil, 2013–2015

Region	Total no. of municipalities^a^	No. of municipalities with remote and deprived populations^b^		No. (%) of physicians allocated^d^
		Total	Enrolled^c^	
North	450	253	228	952 (19.4)
North-east	1795	1287	1077	3547 (72.2)
South-east	1659	73	61	110 (2.2)
South	1200	60	50	204 (4.1)
Mid-west	466	35	34	104 (2.1)
All regions	5570	1708	1450	4917 (100.0)

^a^ Total number of municipalities in each region in 2013.

^b^ Municipalities with remote and deprived populations were those with 20% or more of the population living in extreme poverty (per capita family monthly income below 70 Brazilian reais, equivalent to 19.4 United States dollars) in 2013, and those located in the border areas.

^c^ Of the 1450 municipalities initially enrolled in the programme 39 (2.7%) had dropped out by September 2015.

^d^ Physicians allocated by the programme to municipalities with remote and deprived populations by September 2015.

Sources: Total number of municipalities were obtained from national census data from the Brazilian Institute of Geography and Statistics.^4^ Numbers of municipalities with remote and deprived populations and numbers of physicians allocated were obtained from the Secretariat of Labour Management and Health Education and the Department of Planning and Regulation of the Provision of Health Care Professionals at the Brazilian Ministry of Health, on request, 2015.

**Fig. 2:**
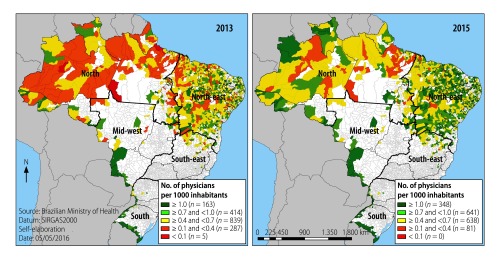
**Density of physicians in municipalities with remote and deprived populations, before and after implementation of the *Mais Médicos* programme in Brazil, 2013 and 2015**

Notes: The *Mais Médicos* programme was implemented in July 2013. Municipalities with remote and deprived populations were those with 20% or more of the population living in extreme poverty (per capita family income below 70 Brazilian reais, equivalent to 19.4 United States dollars) in 2013 or those in the border areas. Of the 1450 municipalities initially enrolled in the programme 39 (2.7%) had dropped out by September 2015. Density of physicians was the number of physicians per 1000 inhabitants in each municipality.

Sources: National Register of Health Institutions, Ministry of Health.^25^ Secretariat of Labour Management and Health Education and the Department of Planning and Regulation of the Provision of Health Care Professionals, Ministry of Health, on request, 2015.

The map for 2013 shows the distribution of the 16 524 physicians already in place and incorporated into primary care teams before the programme started in the 1450 studied municipalities (Fig. 2). After implementation of the programme, 4917 new doctors joined, increasing by 29.8% the availability of medical doctors. There was an increase in the number of physicians per 1000 inhabitants in municipalities with remote and deprived populations. In 2013 there were 292 municipalities with < 0.4 physicians per 1000 inhabitants, and this declined to 81 municipalities in 2015 (decrease of 72.3%). The number of municipalities with ≥ 1.0 physicians per 1000 inhabitants rose from 163 to 348 during the same period, an increase of 113.5% (Fig. 2).

### Primary care coverage

In 2012, there were a total of 6948 primary care teams for a population of 26 742 974 in the enrolled municipalities, a coverage level of 77.9% (100% coverage was 3000 inhabitants per team). By 2015 there were 8038 teams serving 27 929 381 inhabitants, a coverage level of 86.3% (Table 2). Throughout the period 2011–2015, municipalities that did not enrol in the programme had high coverage of primary health care (around 95%), which may explain why they were not interested in enrolling in the programme.

**Table 2:** Primary health-care coverage in municipalities with remote and deprived populations and enrolled or not enrolled in the *Mais Médicos* programme, before and after implementation of the programme in Brazil, 2011–2015

Year	Municipalities enrolled (*n* = 1450)^a^					Municipalities not enrolled (*n* = 258)^a^			
	Total population	No. of PHC teams	No. of inhabitants per PHC team	Coverage,^b^ (%)		Total population	No. of PHC teams	No. of inhabitants per PHC team	Coverage,^b^ (%)
2011	26 538 610	6901	3846	78.0		2 364 218	753	3140	95.5
2012	26 742 974	6948	3849	77.9		2 375 778	754	3151	95.2
2013	27 583 700	7354	3751	80.0		2 437 540	784	3109	96.5
2014	27 762 204	7933	3500	85.7		2 446 769	789	3101	96.7
2015	27 929 381	8038	3475	86.3		2 458 039	777	3163	94.8

PHC: primary health care.

^a^ Of the 1450 municipalities initially enrolled in the programme 39 (2.7%) had dropped out by September 2015. The 258 municipalities not enrolled were eligible for the programme but never enrolled at the time of the study.

^b^ The total population was divided by the number of PHC teams to obtain the number of inhabitants per primary health-care team. This figure was then converted to a percentage, in which 100% coverage was 3000 inhabitants per PHC team, as recommended by the Brazilian Ministry of Health.

Notes: The *Mais Médicos* programme was implemented in July 2013. We studied 1708 municipalities with remote and deprived populations, which were those with 20% or more of the population living in extreme poverty (per capita family monthly income below 70 Brazilian reais, corresponding to 19.4 United States dollars) in 2013, and those located in the border areas.

Sources: DATASUS health portal, Ministry of Health;^26^ Evaluation and Information Management website, Ministry of Social Development.^24^

### Avoidable hospitalizations

In 2012 the percentage of hospitalizations for ambulatory care-sensitive conditions in the municipalities enrolled in the programme was 44.9% (342 908 of the total clinical admissions of 764 342). This decreased to 41.2% (298 566 of 724 921 admissions) by 2015, a decrease of 8.8% (Table 3). By contrast, the rate remained unchanged over this period in the municipalities not enrolled in the programme.

**Table 3:** Rate of potentially avoidable hospitalizations in municipalities with remote and deprived populations, before and after implementation of the *Mais Médicos* programme in Brazil, 2011–2015

Year	Municipalities enrolled (*n* = 1450)^a^			Municipalities not enrolled (*n* = 258)^a^	
	Total no. of clinical admissions	No. (%) of avoidable hospitalizations^b^		Total no. of clinical admissions	No. (%) of avoidable hospitalizations^b^
2011	833 111	376 182 (45.2)		74 549	33 227 (44.6)
2012	764 342	342 908 (44.9)		69 078	30 831 (44.6)
2013	792 385	356 695 (45.0)		69 918	31 710 (45.4)
2014	765 845	332 498 (43.4)		69 247	31 882 (46.0)
2015	724 921	298 566 (41.2)		65 249	29 771 (45.6)

^a^ Of the 1450 municipalities initially enrolled in the programme 39 (2.7%) had dropped out by September 2015. The 258 municipalities not enrolled were eligible for the programme but never enrolled at the time of the study.

^b^ Rate of potentially avoidable hospitalizations was the number of hospitalizations due to ambulatory care-sensitive conditions as a percentage of the total number of clinical admissions. A set of 17 ambulatory care-sensitive conditions were defined in Brazilian Ministry of Health directive MS/SAS no. 221 of 17 April 2008.

Notes: The *Mais Médicos* programme was implemented in July 2013. Municipalities with remote and deprived populations were those with 20% or more of the population living in extreme poverty (per capita family monthly income below 70 Brazilian reais, corresponding to 19.4 United States dollars) in 2013, and those located in the border areas.

Sources: DATASUS health portal, Ministry of Health;^26^ Evaluation and Information Management website, Ministry of Social Development.^24^

### Infrastructure investments

**Fig. 3:**
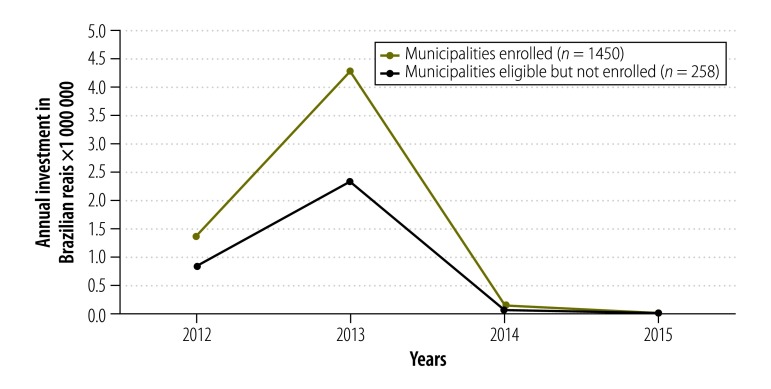
Investment in the construction and renovation of basic health units in municipalities with remote and deprived populations, before and after implementation of the *Mais Médicos* programme in Brazil, 2012–2015

Notes: The *Mais Médicos* programme was implemented in July 2013. Municipalities with remote and deprived populations were those with 20% or more of the population living in extreme poverty (per capita family income below 70 Brazilian reais, equivalent to 19.4 United States dollars) in 2013 or those in the border areas. Of the 1450 municipalities initially enrolled in the programme 39 (2.7%) had dropped out by September 2015. The 258 municipalities not enrolled were eligible for the programme but never enrolled at the time of the study. Data were not available for investments before 2012.

Source: Support unit for strategic management website, Brazilian Ministry of Health.^28^

Investments in basic health units totalled 1.10 billion Brazilian reais in the period 2012–2015, mostly concentrated in 2013, the year the programme was created. These included funds for the construction of 3496 new basic health units and refurbishment of 3417 units. Investments in the municipalities that enrolled in the programme totalled 4275 million Brazilian reais in 2013: 83.4% higher than in those that did not enrol (2331 million Brazilian reais; Fig. 3).

### Medical school places

In 2013,109 (40.7%) of the 268 medical schools in Brazil were in the richer south-east, whereas only 22 (8.2%) were in the north and 25 (9.3%) in the mid-west. From 2013 to 2016 a total of 70 new medical schools were created (public and private), offering 5540 new places for medical students. More than half of the new schools (38 schools; 54.3%) were in the more deprived regions of the north-east, mid-west and north, thus contributing to decreased regional differences.

**Fig. 4:**
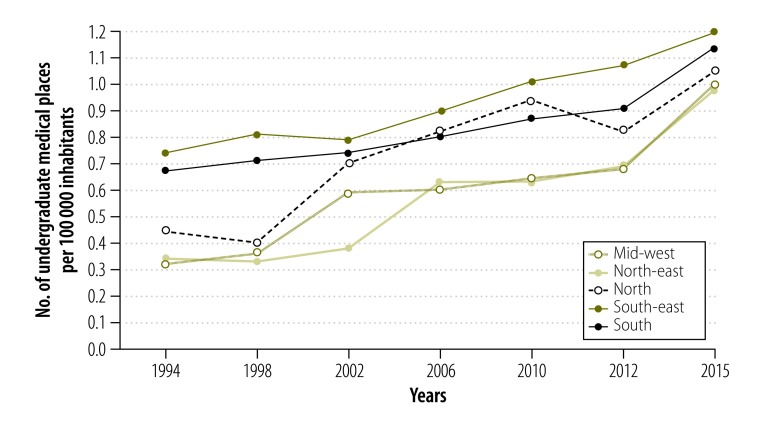
**Number of undergraduate places in medical schools per 100 000 inhabitants, by region of Brazil, 1994–2015**

Note: The *Mais Médicos* programme was implemented in July 2013 in eligible municipalities in all regions.

Sources: Secretariat of Labour Management and Health Education, Ministry of Health.^19^ Medical schools of Brazil website.^29^

We observed a similar pattern with respect to the distribution of undergraduate places in medical schools in 2013. The south-east had 10 639 (43.6%) of the total of 24 401 places and the north-east had 6051 (24.8%) places. There was an increasing availability of undergraduate medical places in Brazil over the years 1994–2015 (Fig. 4). Between 2012 and 2015, however, there was a marked increase in availability of medical school places in all regions of the country, especially the priority areas in the north-east and mid-west. The number of undergraduate places in medical schools in Brazil rose from 0.83 per 10 000 inhabitants in 2012 to 1.07 per 10 000 in 2015.

## Discussion

The *Mais Médicos* programme provides physicians to areas where deprived populations are living in conditions of extreme poverty. Working towards a goal of guaranteeing the right to universal health care, the programme facilitates access to health services for people who have seldom, or never, had access to that right.^30^^,^^31^

The municipalities that we studied were remote, and in the case of the Amazon region may be accessible only by boat or aeroplane. These regions face problems of recruitment and retention of physicians, primarily because they are far from large urban centres.^32^^,^^33^ Previous interventions such as PROVAB^34^ – based on successes in other countries – did not achieve the desired results, especially in the remote areas, due to low participation by Brazilian physicians. The *Mais Médicos* programme was set up to make more effective changes in the provision and attachment of physicians to primary health care. There was some resistance at first, especially from the Federal Medical Council, which headed a petition in the Supreme Court alleging that the programme was unconstitutional.^35^^,^^36^ The request was not approved, but disputes continue, due to controversy about hiring foreign doctors. The general public, however, has recognized the need for more physicians as many areas in Brazil have had insufficient numbers of physicians for long periods.^34^ Opinion polls conducted by the Brazilian National Transport Confederation, disclosed that the programme was approved by 49.7%, 73.9% and 84.3% of the population of 2022 respondents in July, September and November of 2013 respectively.^36^

The data from our study showed that the programme succeeded in providing greater numbers of primary health-care physicians to remote and deprived populations and an increase in primary health-care coverage.

Introduction of the programme also coincided with important changes in health care, such as the reduction in hospital admissions due to ambulatory care-sensitive conditions, which could reflect positive changes in primary health-care practices. The programme could also be linked to mid- and long-term measures, such as increased investments in basic health units, infrastructure and equipment. An increase in the number of medical school places in Brazil was recorded in the period after implementation of the programme, resulting in a more equitable geographical distribution of places.

Similar programmes to expand professional training have been conducted in other countries with remote areas.^37^^,^^38^ The need to expand the number of medical courses in Brazil was based on the low supply of undergraduate medical places, which was 0.8 per 10 000 inhabitants in 2011, half that of other countries with universal health systems, such as the United Kingdom of Great Britain and Northern Ireland (1.6 per 10 000 inhabitants).^39^ In countries such as Australia, Canada and the United States of America, training programmes to serve populations in remote and rural areas where it was difficult to recruit and retain physicians were implemented in medical schools in the 1960s.^11^ Medical schools adopted measures to promote the selection of students from rural communities and enacted curricular reforms that provided theoretical and practical learning, with a focus on primary health care.^12^ In Australia, for example, the Rural Clinical Training and Support programme finances medical training aimed at remote and rural areas.^15^

A limitation of our study is that the short time elapsed between the before and after evaluations precludes analysis of its impact on the health of the population. Further studies are needed to assess the impact of the programme on the population in Brazilian municipalities with extreme poverty.

Keeping up the recruitment and provision of physicians in the future is a major challenge for Brazil. The first phase of the programme had no long-term perspective. It was a temporary policy, effective for three years according to the law passed in October 2013. A new provisional measure to extend the temporary foreign exchange visa scheme for physicians for three more years was approved in September 2016. The health ministry has indicated that the programme will continue for this period. The provision of physicians to remote areas in the long term, however, will require measures such as the creation of a career in the public system that will provide greater job security and access to continuing education.^40^ On the other hand, this will not be possible if the chronic underfunding of the national unified health system is not solved.^41^ Less than one-third of physicians recruited by the programme up to September 2015 were Brazilians. Maintaining the programme in Brazil with foreign medical collaboration may be essential until sufficient new physicians have graduated to meet the need of all municipalities.

In conclusion, the *Mais Médicos* programme was successful in the immediate provision of physicians to work in primary health care with the most remote and deprived populations. In the longer term the programme assured investments, refurbishment and construction of basic health units and expansion of the network of medical schools, following a more equitable geographical distribution. Similar strategies could be established in other countries with a shortage of physicians, especially underdeveloped nations, to contribute towards achieving universal health access and coverage for all as proposed by the SDGs.
